# A Practical Treatise on Inflammation of the Uterus; Its Cervix and Appendages, and on Its Connection with Uterine Disease

**Published:** 1853

**Authors:** 


ART. X.—*A Practical Treatise on Inflammation of the Uterus; its Cervix and Appendages, and on its Connection with Uterine Disease.* By JAMES HENRY BENNET, M.D. Philadelphia: Blanchard & Lea. 1853.

The author of this treatise says, in his preface, that the doctrines which it sets forth have made great progress since the publication of previous editions, and that from all parts of the globe he has received testimonies of approbation from those who have made actual investigation of the subject. Few affections have been investigated with more success during the last few years than those to which Dr. Bennet has specially devoted himself, and the reason for it is obvious: they have been examined by physical means sufficient to reveal their character. We owe our progress in this department of pathology and practice, to the introduction of instruments as means of diagnosis. The practical results of this step have been more striking than those which have flowed from the use of the stethoscope in diseases of the chest. The new method has revolutionized the practice in uterine diseases. It has given a certainty to our remedial efforts in such complaints which it were impossible to attain by any other method.

Dr. Bennet expresses surprise that this mode of examining the uterus in its morbid conditions should have been so long overlooked by the profession. It is more surprising that, having been introduced and so beneficially applied, it should meet with the determined opposition of so many practitioners. Inspection of the uterus is, even now, practiced by comparatively few physicians, and by many it is regarded, not merely with aversion, but with hostility. The procedure is denounced as immoral. It is alleged that it offends, and will blunt, the delicacy of the sex.

Such practitioners, we think, are mistaken. The argument of immorality would apply with quite as much force to the practice of midwifery, by men, and has, indeed, been urged by the advocates of female medical education. No such moral deterioration results, in our belief, from either practice. Will any one assert that those women who have been the subjects of male-midwifery or of the speculum, are less pure than those who have had only female accoucheurs, or have escaped the necessity of medical attendance altogether? But we cannot believe that this objection to the speculum is entitled to grave consideration.

We were present, not long since, at a discussion on this subject, and were much impressed by a statement made by Prof. Miller, whose experience in the use of the speculum is known to have been very great. He said that he had within a few years past had patients from nearly every county in Kentucky, laboring under disease of the cervix uteri, and that when one came to him he was pretty sure that she would soon be followed by another. And he added, that his success in treating these affections, had only been such since he began to employ the speculum as a means of diagnosis. The positive evidence of one such practitioner, founded upon ample observation and experience, we take to be worth more than the speculative objections of a thousand men who have made no trials with the instrument. Any one who has read the standard works on uterine diseases, published only a few years back, and has realized how vague is the instruction they convey on many subjects, will hail any new light that may be thrown upon these troublesome disorders. One of the best of these works is confessedly the treatise of Sir Charles Clarke on Female Discharges, and yet, as every reader knows, it fails to notice one of the most frequent causes of such a condition—inflammatory ulceration of the cervix. What, in truth, was known of such affections before the inauguration of this new mode of diagnosis? Our remedies were directed in the dark, because we had no precise idea of the nature of the affection. We prescribed for symptoms, while the cause of the complaint remained undiscovered, and so we went on, too often trying, without avail, the whole round of alteratives, styptics, and astringents. But since the adoption of instrumental examination we have been able to fix the seat of the disease, and to reach it by efficient remedies.

The use of the speculum in diseases of the uterus is not only valuable as a guide to the application of powerful agents when they are demanded, but as warning the practitioner against their use when not required. Dr. Bennet says on this point:—

"Not only is it *possible* to treat successfully non-ulcerated inflammation of the cervix, when slight, and of recent date, merely by emollient and astringent injections, rest, and attention to general health, without having recourse to instrumental examination, or to means of treatment requiring instrumental interference, but even slight ulcerations, unaccompanied by general inflammatory hypertrophy, and unattended with disease of the urinary canal, will sometimes give way under the influence of these means. In order to establish this fact, after ascertaining with the speculum the presence of a superficial ulceration of this description, I have repeatedly thus treated the patient, without using any other local application to the ulcerated surface, and have found the inflammation diminish, the ulceration decrease, and at last cicatrice.

It is only, however, in cases of slight ulceration, unaccompanied by general hypertrophy or by cervical disease, a rare condition, that emollient and astringent injections alone succeed; and even in these exceptional cases the treatment cannot be depended upon. Moreover, the recovery, when it does take place, is so much more tedious than when cauterization of the ulcerated surface is resorted to, that I never feel authorized to recommend its adoption, if the existence of ulceration has once been instrumentally recognized; as long as it is only suspected, and there does not seem sufficient grounds to warrant an examination, the employment of these local means of treatment, is, however, the rational course."

The remarks of Dr. Bennet on ulceration of the cervix uteri, and the use of cauteries in such cases, are highly interesting. We quote one passage :—

"A few years ago, in this country, ulcerative disease of the uterine neck was seldom detected, even by the most eminent uterine practitioners of the day. In a large proportion of the chronic cases of this description, for which I was then consulted in private practice, the very existence of the inflammatory ulceration from which the patient had been suffering for many years had not been even suspected, notwithstanding many valued opinions had been taken. Since the attention of the profession was directed, in the first edition of this work, to the frequency of this form of disease, and since the doctrines therein promulgated have been adopted and acted upon by many leading practitioners, I have observed fewer instances of non-detection of ulcerative disease. I am still, however, continually witnessing cases in which ulceration has thus been imperfectly recognized and treated, the external or cervical ulceration only having been attended to, and the internal ulcerative element remaining unperceived. This error is committed in Paris as well as in this country. I never recollect seeing the cervical cavity examined, as I now invariably examine it, when I held office in the Paris hospitals ; and in what has been written by French pathologists on uterine diseases, there is no evidence of their being acquainted with the fact of ulceration so frequently penetrating and lurking in the cavity of the cervix. On the contrary, they mistake for indications of internal metritis the discharges which exist when the cervical cavity is inflamed or ulcerated.

The agents which may be used for cauterization of the cervix are varied. The principal are the nitrate of silver, the mineral acids, and more especially the acid nitrate of mercury, potassa fusa and potassa cum calce, and the actual cautery. We will successively examine each of these agents.

The most generally employed, and at the same time the least energetic caustic, is the nitrate of silver. Indeed, it scarcely deserves the name of caustic, so superficial is its action. When freely applied in substance to the granulations which cover the ulcerated surface, it forms a white film or eschar, the thickness of which, when it falls, is seldom greater than that of a piece of drawing-paper. This eschar is thrown off either entire or piece-meal, about the third or fourth day. On the latter day, the surface to which the solid nitrate of silver has been applied, is generally found red, irritable, and bleeding. On the fifth day, however, all apparent irritability and tendency to bleed disappear, and by this or the following day, the amount of benefit to be obtained from the application is generally ascertained, the ulceration seldom improving subsequently. If left to itself, indeed, it soon again becomes morbidly irritable, and occasions pain and sympathetic recreation on the general system. When a solution of nitrate of silver is used, these effects are obtained in a shorter space of time, and it may consequently be applied at shorter intervals than every fifth or sixth day, the period which should be allowed to elapse between the applications of the solid nitrate. In some cases, a strong solution thus employed may be more beneficial than the solid nitrate, but as it entails a more frequent use of instrumental means, the great drawback in the treatment of these diseases, I generally confine myself to the use of the solid caustic.

The periodical application of the nitrate of silver to the ulceration often suffices to bring on healthy action, and to cause the ulceration, if small and recent, to heal in a few weeks. Even when it is covered with fungous, livid granulations, and secretes an abundant sanguineo-muco-purulent discharge, the solid caustic, freely applied, generally arrests the exudation of blood, and brings the ulcer to a clean, healthy, and comparatively dry state after two or three applications; although it is seldom sufficiently powerful to modify the vitality of such a diseased surface, so as to produce cicatrization. In these cases, however, the solid nitrate of silver is a most valuable agent, as it is applicable in a stage of the disease when other and more powerful remedies can scarcely be used. Owing to the very limited cauterizing powers of the nitrate of silver, it may be employed without the precautions which the more powerful caustics imperatively require. Its being dissolved to a considerable extent by the blood and muco-pus which freely exude from these ulcerations, is of no consequence; so far from doing harm to the surrounding tissues, if it runs on and touches them, it acts, on the contrary, beneficially, as a powerful astringent, if they are at all inflamed, which they generally are. When applied to a non-ulcerated, mucous surface, it merely seems to produce a white film or epithelial eschar, the falling of which is never followed by ulceration or excoriation, all evidence of its having been applied disappearing in a few days.

If the ulceration penetrates into the cervical cavity, the solid nitrate of silver may be pushed into it as far as it will enter, or a camel-hair pencil, loaded with a saturated solution, may be used in the same way. There is no fear, as we have seen, of penetrating too far, as the cervical canal is only sufficiently dilated to admit the brush, or the caustic cylinder, in the region to which inflammatory action extends. Beyond the point where inflammation ceases, the natural and healthy coarctation of the cervical canal will prevent their passing. I prefer the brush when the inflammation penetrates very far, lest the stick of caustic should break. This has occurred to me more than once, but I have never had any difficulty in extracting the fragment, either by means of the speculum forceps, the end of which I have had purposely made small, or of the uterine sound. Thence the necessity of examining the piece of caustic that has been used, when it is withdrawn, in order to see that it is entire."

This is emphatically a work for the practitioner of medicine; who can hardly pass a day without meeting with cases demanding knowledge which it contains. We can conscientiously recommend it to our brethren in the most decided terms.

